# *Pseudomonas aeruginosa* adaptation and diversification in the non-cystic fibrosis bronchiectasis lung

**DOI:** 10.1183/13993003.02108-2016

**Published:** 2017-04-27

**Authors:** Yasmin Hilliam, Matthew P. Moore, Iain L. Lamont, Diana Bilton, Charles S. Haworth, Juliet Foweraker, Martin J. Walshaw, David Williams, Joanne L. Fothergill, Anthony De Soyza, Craig Winstanley

**Affiliations:** 1 Institute of Infection and Global Health, University of Liverpool, Liverpool, UK; 2 Dept of Biochemistry, University of Otago, Dunedin, New Zealand; 3 Dept of Respiratory Medicine, Royal Brompton Hospital, London, UK; 4 Cambridge Centre for Lung Infection, Papworth Hospital, Cambridge, UK; 5 Dept of Respiratory Medicine, Liverpool Heart and Chest Hospital, Liverpool, UK; 6 Institute of Integrative Biology, University of Liverpool, Liverpool, UK; 7 Institute for Cellular Medicine, Newcastle University, Newcastle-upon-Tyne, UK; 8 Adult Bronchiectasis Service, Freeman Hospital, Newcastle-upon-Tyne, UK; 9 These authors contributed equally; 10 These authors contributed equally

## Abstract

To characterise *Pseudomonas aeruginosa* populations during chronic lung infections of non-cystic fibrosis bronchiectasis patients, we used whole-genome sequencing to 1) assess the diversity of *P. aeruginosa* and the prevalence of multilineage infections; 2) seek evidence for cross-infection or common source acquisition; and 3) characterise *P. aeruginosa* adaptations.

189 isolates, obtained from the sputa of 91 patients attending 16 adult bronchiectasis centres in the UK, were whole-genome sequenced.

Bronchiectasis isolates were representative of the wider *P. aeruginosa* population. Of 24 patients from whom multiple isolates were examined, there were seven examples of multilineage infections, probably arising from multiple infection events. The number of nucleotide variants between genomes of isolates from different patients was in some cases similar to the variations observed between isolates from individual patients, implying the possible occurrence of cross-infection or common source acquisition.

Our data indicate that during infections of bronchiectasis patients, *P. aeruginosa* populations adapt by accumulating loss-of-function mutations, leading to changes in phenotypes including different modes of iron acquisition and variations in biofilm-associated polysaccharides. The within-population diversification suggests that larger scale longitudinal surveillance studies will be required to capture cross-infection or common source acquisition events at an early stage.

## Abstract 2

***P. aeruginosa* co-infections occur in bronchiectasis; bacterial populations both adapt and diversify by mutation** http://ow.ly/fMhj308zUv0

## Introduction

Bronchiectasis is a chronic, progressive respiratory disease associated with irreversible widening of the bronchi [1]. Recent data suggest that in the UK, incidence rates in females and males have risen to 35.2 and 26.9 per 100 000 person-years, respectively [2]. In the USA, the prevalence of adult bronchiectasis has been estimated at 52 in 100 000 people, with higher prevalence among females and older individuals [3]. Persistent *Pseudomonas aeruginosa* lung infections of bronchiectasis patients, occurring in ∼30% of cases, are associated with poorer outcomes and premature mortality [4, 5].

The study of chronic *P. aeruginosa* lung infections has focused on cystic fibrosis (CF)-associated bronchiectasis, where patients are diagnosed, monitored and subjected to antibiotic therapy from a very early age. This contrasts with non-CF bronchiectasis patients, who present at a much older age and often have a shorter history of therapeutic interventions. Hence, bacterial isolates from non-CF bronchiectasis patients exhibit less resistance to antibiotics compared to isolates from adult CF patients [6]. Previous studies have characterised the evolution of *P. aeruginosa* during chronic lung infections in CF patients [7, 8]. High-resolution analyses have revealed extensive heterogeneity within *P. aeruginosa* populations in the CF lung [9–12], including the co-existence of multiple divergent lineages [13].

In CF, a number of transmissible strains of *P. aeruginosa* have been identified, leading to the introduction of measures to control cross-infection [14]. The study of *P. aeruginosa* in relation to non-CF bronchiectasis is less advanced. In our single-centre study of 50 *P. aeruginosa* isolates from 40 bronchiectasis patients using molecular typing, there was no compelling evidence for cross-infection or a dominant clone [15]. However, whole-genome sequence analysis of multiple bronchiectasis isolates has not been performed. Here, we report the use of genomics to assess the diversity of *P. aeruginosa* strains causing infections in non-CF bronchiectasis across multiple UK centres, to identify multistrain infections, and to look for evidence for cross-infection or common source acquisition. In addition, we characterise adaptive mutations and present evidence for within-population divergence during *P. aeruginosa* chronic lung infections of bronchiectasis patients.

## Methods

### Patients and bacterial isolates

The 189 *P. aeruginosa* isolates used in this study (online supplementary table S1) were isolated from sputum samples obtained from 93 patients with bronchiectasis and chronic *P. aeruginosa* infection (defined as two or more positive respiratory tract cultures in the preceding 12 months) attending 16 adult bronchiectasis centres throughout England and Wales. These included isolates collected as part of a multicentre nebulised antibiotic trial [16], where patients were enrolled within 21 days of completing a course of antipseudomonal antibiotics for an exacerbation. Additional isolates from Newcastle (n=8) and Liverpool (n=53) were collected during observational studies. The methodology used for isolating *P. aeruginosa* from patient sputum samples is described in the online supplementary material.

For 24 patients, sets of isolates (two or more) from the same sample were analysed to look for evidence of multilineage infections. For three of these patients (patients 147–149), sets of 14 or 15 isolates from a single sample were sequenced for higher resolution analysis of within-population heterogeneity. For some analyses, to avoid biases arising from inclusion of multiple clonal genomes from the same patient, a subset of 99 genomes from 91 patients was used. This subset consisted of one randomly selected genome per clonal lineage per patient (online supplementary table S1). We use the term “clonal lineage” to describe isolates with shared multilocus sequence type (MLST) profile and clustering according to core genome single nucleotide polymorphism (SNP)-based phylogeny.

### DNA preparation and whole-genome sequencing

Details of the extraction of genomic DNA from *P. aeruginosa* isolates, library preparation and whole-genome shotgun sequencing using Illumina (San Diego, CA, USA) short-read sequencing technology are given in the online supplementary material. The European Nucleotide Archive accession number for the study is PRJEB14952.

Methods used for genome sequence assembly, extraction of MLST data, phylogenetic reconstruction using the core genome and variant calling by mapping to the genome of PAO1 [17] to identify SNPs or small insertions or deletions are described in the online supplementary material.

### Identification of large deletions and virulence factor genes

Genome sequences were aligned to the reference genomes *P. aeruginosa* PAO1 (NC_002516 [17]) and *P. aeruginosa* LESB58 (FM209186 [18]) and large clone-specific deletions (≥10 kb) were identified using the BLAST Ring Image Generator (BRIG) [19]. The boundaries of deletions were determined by aligning the genome sequences with the *P. aeruginosa* PAO1 genome using Mauve [20], implemented as part of the Geneious package (www.geneious.com). The presence and absence of virulence factor genes in genome assemblies was determined using Blastable (www.github.com/bawee/blastable). The *Pseudomonas* genome database (beta.pseudomonas.com) [21] was used to facilitate analysis of gene function.

## Results

### Diversity of *P. aeruginosa* non-CF bronchiectasis isolates and evidence for *P. aeruginosa* multilineage co-infections

**FIGURE 1:**
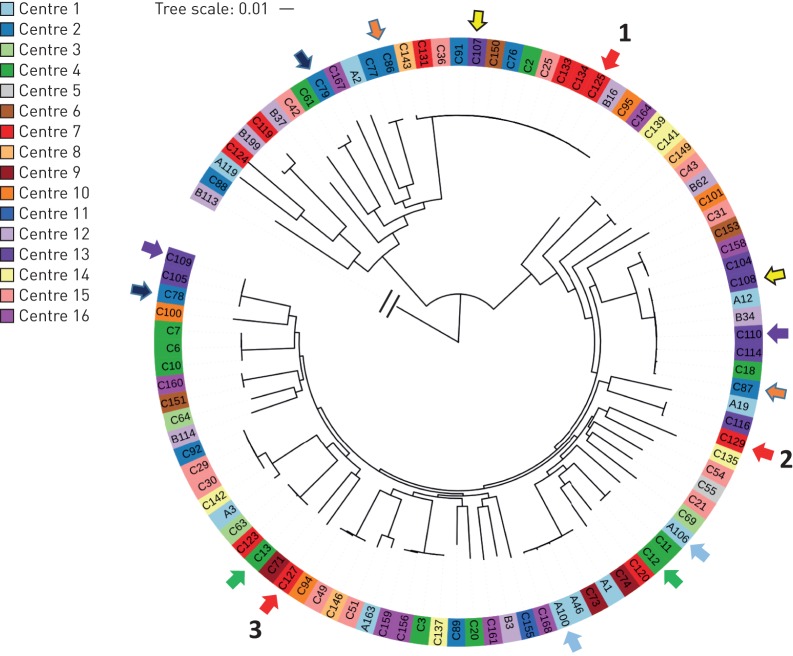
Evidence for multilineage co-infections in seven patients. A core genome single nucleotide polymorphism phylogeny is shown for the subset of 99 isolates, confirming that all but one isolate (B113) clusters into one of two major groups. Each bronchiectasis centre is represented by a different colour. Arrows sharing the same colour indicate isolates that were obtained from the same patient. The three isolates from the same patient 92 sample are numbered 1–3.

Core genome SNP phylogenetic analysis alongside a collection of 331 *P. aeruginosa* isolate genomes from diverse clinical sources [22] indicated that the bronchiectasis isolates were widely distributed (online supplementary figure S1). From the 189 isolates, it was possible to extract complete MLST profiles for 160 (online supplementary tables S1 and S2), with the most widespread sequence types (STs) being ST-253 (PA14-like [23]; 14 patients, eight centres), ST-179 (seven patients, four centres), ST-17 (clone C [23]; five patients, three centres), ST-252 (four patients, four centres) and ST-260 (four patients, three centres). Using core genome SNP phylogeny, previous studies have subdivided the wider *P. aeruginosa* population into two major groups (group I, which includes strain PAO1, and group II, which includes strain PA14) and one minor group of mostly unrelated clonal lineages [24, 25]. Of a subset of 99 genomes consisting of one randomly selected genome per clonal lineage per patient, 71 were located in group I and 27 in group II (figure 1). Based on a combination of MLST genotype and core genome SNP phylogeny, of the 24 patients from whose samples multiple isolates were examined, there were seven examples of multilineage infections. In one patient (patient 92), three distinct clonal lineages of *P. aeruginosa* were identified. In patients 42, 72, 73, 84, 85 and 148 there were two co-existing lineages (figure 1).

### Evidence of shared lineages causing infections in different patients attending the same centre

**FIGURE 2:**
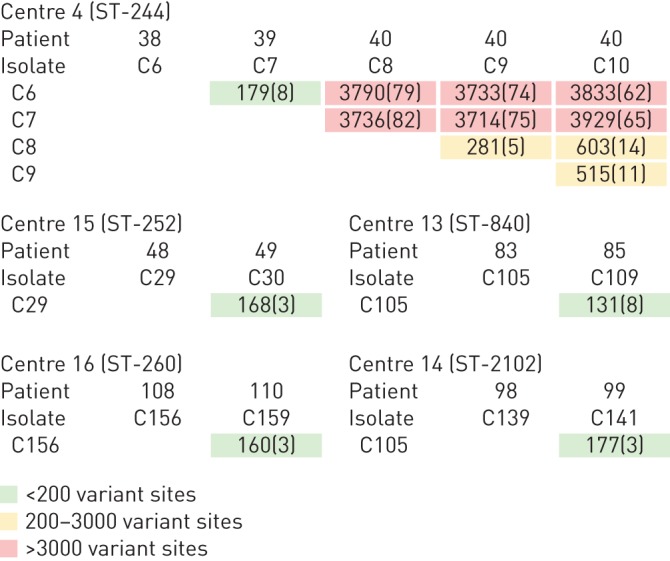
Example pairwise comparisons between isolates sharing the same clonal lineage that were isolated from more than one patient attending the same centre. The number of single nucleotide polymorphism variations are indicated, with the number of small insertion and deletion variations shown in brackets. Full details are shown in online supplementary table S3. The five examples where isolates shared <200 variant sites are highlighted in green. All isolates of ST-244 from patients attending centre 4 were compared, with similarity graded according to variant sites.

The core genome SNP phylogeny identified a number of examples where closely related clonal lineages were isolated from more than one patient attending the same centre (online supplementary table S3). In order to obtain a higher resolution comparison, these isolates were analysed using pairwise comparisons across their entire genomes (online supplementary table S3), identifying five instances where the genomes of isolates from different patients attending the same centre varied at <200 sites (C6/C7, C29/C30, C105/109, C139/C141 and C156/C159; figure 2). This level of genome similarity is greater than in some pairwise comparisons of contemporary isolates of the same lineage from the same sputum sample (online supplementary table S3; from 184 variant sites (C110/C111) to >750 variant sites (C125/C126)).

**FIGURE 3:**
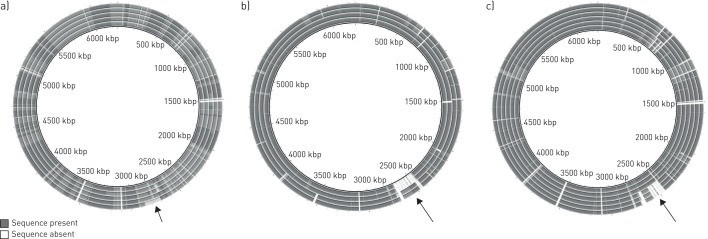
Examples of alignment of genomes of bronchiectasis strains with that of reference strain *Pseudomonas aeruginosa* PAO1. Sequences identified as present (dark grey) or absent (white) in the genome of PAO1 are indicated. a) Isolates of the same lineage (ST-253) from the same patient. From innermost to outermost: C95, C97, C98, C99 and C96. A deletion present in isolate C96 only is highlighted (arrow). b) Pairs of isolates (from innermost to outermost: C6 and C7; and C156 and C159) that both share the same clonal lineage but are from different patients attending the same hospital. Isolates C6 and C7 share a large deletion and isolates C156 and C159 share a smaller overlapping deletion (online supplementary table S4), as indicated (arrow). c) Isolates of different lineages from the same patient. From innermost to outermost: A77, A80 and A85 (all ST-175); and A78, A81 and A82 (all ST-17). A large deletion present in the ST-17 isolates is indicated by an arrow. The figures were generated using the BLAST Ring Image Generator [19].

The draft genome sequences of the subset of 99 bronchiectasis isolates were examined for the presence of large (>10 kb) deletions (figure 3). A total of 36 different deletions (25 >100 kb), ranging in size from 11 to 300 kb and representing independent genetic events, were identified (online supplementary table S4). These were distributed across 28 genomes in the 99-member genome subset. Most genomes had only one deletion, although two (C54 and C164) had three deletions and four (A119, C4, C85 and C119) had two. In most cases, isolates of the same clonal lineages from the same patient shared the same deletions. However, in patients 45, 55, 79 and 92 not all isolates of the same lineage had the same deletion. The genomes of isolate pairs C6/C7, C29/C30, C105/109, C139/C141 and C156/C159, which are from different patients but vary at <200 sites (table 1), were indistinguishable by BRIG analysis (example shown in figure 3b).

**TABLE 1:** Summary of genomic diversity observed within the same clonal lineage of *Pseudomonas aeruginosa* in individual patients

	**Isolates**	**SNPs**	**INDELs**
**Patient 147**	15	336.35; 261.00 (88–640)	15.20; 14.00 (0–35)
**Patient 148 (ST17)**	4	451.50; 482.50 (159–654)	23.83; 25.50 (6–34)
**Patient 148 (ST175)**	11	195.45; 179.00 (79–403)	9.27; 5.00 (0–36)
**Patient 149**	14	209.01; 206.00 (68–327)	11.40; 10.00 (3–28)

Data are presented as n or mean; median (range). The number of single nucleotide polymorphisms (SNPs) and small insertion and deletion (INDEL) differences between the genomes of contemporary isolates from single sputum samples are presented.

### Genomic diversity of isolates within patients can be similar to diversity between patients

In order to further assess the within-patient diversification exhibited by *P. aeruginosa* populations, larger sets of isolates from single sputum samples were analysed for three patients: 147 (15 isolates), 148 (15 isolates) and 149 (14 isolates) (table 1). For two of these patients, the *P. aeruginosa* population comprised a single clonal lineage. For patient 148, two distinct clonal lineages were identified and these two sets of isolates were analysed separately. In all four isolate sets analysed, the maximum pairwise SNP variations between two isolates of the same lineage was >300, with a median of ≥79 (table 1), indicating the occurrence of within-patient diversification.

### Loss-of-function mutations and deletions identified in multiple isolates

We used variant calling approaches to identify independent occurrences of loss-of-function mutations within the subset of 99 bronchiectasis isolate genomes. This yielded a number of examples of genes with known functions carrying independent loss-of-function mutations in multiple isolates (table 2 and online supplementary table S5). These include genes linked to mucoidy, virulence, osmoprotection, biofilm formation, motility, DNA repair and antimicrobial resistance (table 2). The genes encoding all three components of the MexAB-OprM efflux pump appear among the most common loss-of-function mutations. In addition, multiple isolates carried loss-of-function mutations in genes encoding regulators (including *lasR*, *algU*, *fleR* and *vfr*). Among the 99 bronchiectasis isolates, the number of genes with loss-of-function mutations as listed in table 2 ranged from zero to six (online supplementary figure S2 and table S6).

**TABLE 2:** Loss-of-function mutations occurring in multiple isolates

**Gene**	**PAO1 gene number**	**Independent occurrences of a mutation n**	**Function/comment**
***mexB***	PA0426	16	Transporter from MexAB-OprM efflux pump, antibiotic resistance, virulence
***mucA***	PA0763	13	Anti-σ factor, mutations can lead to mucoidy
***betT2***	PA5291	9	Transporter, uptake of small molecules such as choline and glycine betaine, contributing to growth *via* phosphatidyl choline metabolism and osmoprotection
***bifA***	PA4367	7	Cyclic-di-GMP phosphodiesterase, inversely regulates biofilm formation
***mexA***	PA0425	7	Membrane fusion protein from MexAB-OprM efflux pump, antibiotic resistance, virulence
***pcoA***	PA2065	7	Copper resistance
**PA4469**	PA4469	7	Hypothetical protein encoded by a gene in same operon as and upstream of *sodM* (superoxide dismutase; response to oxidative stress)
***rbdA***	PA0861	7	Cyclic-di-GMP phosphodiesterase, modulation of biofilm dispersal, negative regulation of Pel production
***pilJ***	PA0411	6	Methyl-accepting chemotaxis receptor-like protein involved in twitching motility and biofilm formation
***oprM***	PA0427	6	Outer membrane protein from MexAB-OprM efflux pump, antibiotic resistance, virulence
***oprF***	PA1777	6	Major porin, biofilm formation
***chpA***	PA0413	5	Chemotaxis-like chemosensory protein involved in twitching motility
***fimV***	PA3115	5	Peptidoglycan-binding protein, promotes type IV pilin assembly, twitching motility
***ladS***	PA3974	5	Sensor kinase, implicated in switch between acute and chronic infection
***mutL***	PA4946	5	Mismatch repair system, DNA repair, mutation can lead to mutator phenotype
***gmd***	PA5453	5	GDP-mannose 4,6-dehydratase,
***mexS***	PA2491	5	Mutations promote MexT-dependent *mexEF-oprN* expression and multidrug resistance
***pchE***	PA4226	5	Pyochelin synthesis
**PA0054**	PA0054	5	Hypothetical protein

Only mutations predicted to lead to loss-of-function were included (*i.e.* introduction of a stop codon, or a frame-shift mutation). The number of independent mutations indicates the number of isolates carrying unique mutations in the listed gene. Those genes where the number of independent occurrences of a mutation was equal to or greater than five are shown.

Hypermutability is a common trait among CF isolates of *P. aeruginosa*. Of the 99 panel isolates, 11 carried loss-of-function mutations in the DNA mismatch repair genes *mutS* or *mutL* (online supplementary table S1). All but two of these were confirmed as having the hypermutable phenotype.

An alignment of all of the genomes containing deletions >10 kb relative to the genome of strain PAO1 revealed a strikingly nonrandom distribution, with 30 of the 36 deletions lying within the 1.9- to 2.8-Mb portion of the strain PAO1 genome. Genes within this region include the *psl* genes, encoding an extracellular polysaccharide [26], genes encoding the siderophore pyoverdine and genes encoding a type VI secretion apparatus [27].

Next, we specifically examined one representative of each of the 99 clonal lineages for the presence or absence of genes associated with pathogenicity (online supplementary table S6). 23 of these genomes lacked one or more of the *psl* genes. In contrast, all of the genomes contained all of the *alg* genes required for making alginate and the *pel* genes required for making Pel exopolysaccharide. 11 of the genomes lacked genes required for synthesis of pyoverdine, with nine of these also lacking an *fpvA* receptor gene for uptake of ferripyoverdine, although the genes required for synthesis of an alternative siderophore, pyochelin, were present in all cases. In addition, 11 of the genomes lacked two or more genes of the type VI secretion system (PA2360 (*hsiA3*)–PA2373 (*vgrG3*)) (online supplementary table S6). These findings are consistent with the occurrence of deletions of the region of the genome containing Psl, pyoverdine and type VI secretion genes in multiple isolates, although in some isolates smaller deletions (<10 kb) were detected.

## Discussion

We used whole-genome sequencing to obtain a cross-section of the diversity of *P. aeruginosa* strains causing infections in bronchiectasis in the UK. Our data suggest that the distribution of *P. aeruginosa* lineages found among the bronchiectasis isolate collection broadly represents what is present in the global *P. aeruginosa* population. In contrast to CF [14], we found no data to suggest that there is a widespread transmissible strain among the UK non-CF bronchiectasis community. However, our study did not include large numbers of patients from individual centres. Lineages such as PA14-like and clone C, that are naturally more abundant in nature [23], were among the most abundant in the bronchiectasis collection. Because some lineages are naturally more abundant, their occurrence (based on MLST) in multiple patients is not necessarily indicative of common source or cross-infection. Whole-genome sequencing offers higher resolution than methods such as MLST, allowing us to address this issue.

In a previous comparison of paired isolates from patients within the same bronchiectasis centre, in most patients (nine out of 10) the two isolates shared a common genotype, with one patient found to be infected with two strains simultaneously [15]. In this study, of 24 patients from whose samples multiple isolates were examined, seven had multilineage infections. Similar multilineage infections have also been reported in CF, generally associated with children [28]. In addition, a number of studies in CF have also demonstrated the phenotypic [9, 11, 12] and genomic [10, 13, 29] diversification of single-lineage *P. aeruginosa* populations in the CF lung. Here, we show for the first time that similar diversification occurs during infections of non-CF bronchiectasis patients. Both the prevalence of multilineage infections and the diversification that occurs during the infection process emphasise the need to be cautious when interpreting the analysis of sputum samples based on single isolates of *P. aeruginosa*.

We found several examples of isolates from patients attending the same centre that not only shared the same clonal lineage, but also differed genomically by <200 sites. Genomic variations between isolates from the same patient sample revealed similar, and in some cases higher, levels of variation. The occurrence of isolates with very high genetic relatedness in different patients strongly implies that there has been common source acquisition or cross-infection. The extent of the nucleotide variations differentiating two isolates will be dependent upon 1) the length of time since the transmission event and 2) the rate of mutation of the *P. aeruginosa* population during the infection. Further studies will be needed to better define the role of cross-infection or common source acquisitions in this patient group.

There was clear evidence for bacterial adaptation to the lung environment by the accumulation of mutations and deletions, including loss-of-function mutations in genes identified previously as being commonly mutated in CF, such as *mucA* (mucoidy) and *lasR* (quorum sensing). However, it is worth noting that mutations in genes encoding some of the regulators highlighted in previous CF studies (*mexT*, *retS*, *exsD* and *ampR*) were observed either infrequently (two *mexT* and two *ampR* mutants) or not at all (online supplementary table S5). Mutations in global regulators potentially affect numerous processes. In CF, the pathoadaptive genes identified in different studies have varied, suggesting that there are multiple routes to adaptation to the CF lung [7, 8], a scenario which is likely to apply also to non-CF bronchiectasis.

Loss-of-function mutations in genes encoding the MexAB-OprM efflux pump were common among the bronchiectasis isolates. Although generally thought of as a multidrug efflux system important for antibiotic resistance, this system has been implicated in virulence [30]. Hence, although it may seem counterintuitive that *P. aeruginosa* should adapt by losing an antibiotic resistance-related efflux pump, it may be that the driver for selection is related to a function other than antibiotic efflux. In contrast, the loss-of-function mutations in *mexS* can be linked directly to antibiotic resistance, since mutations in *mexS* promote upregulation of the MexEF-OprN MDR efflux pump [31].

The prevalence among non-CF bronchiectasis isolates of deletions in a specific genomic region encoding pyoverdine and Psl polysaccharide was higher than in a dataset of 331 *P. aeruginosa* clinical isolate genomes [22], where 22 genomes lacked one or more *psl* genes, only three lacked one or more of the pyoverdine synthesis genes and only one did not have an *fpvA* receptor gene. *P. aeruginosa* can utilise multiple pathways for iron acquisition [32]. During chronic lung infections in CF, *P. aeruginosa* adapts by favouring the heme utilisation route for iron acquisition rather than the pyoverdine siderophore system [33]. Our observations suggest a similar adaptation in non-CF bronchiectasis.

In order to protect itself from hostile environmental conditions or host defences *P. aeruginosa* can produce three exopolysaccharides contributing to biofilm formation: alginate, Psl and Pel [26]. It has been suggested that Psl is a key surface attachment determinant [34], whereas in the CF lung free-floating biofilm structures may be more important [35]. Other mutations favouring the production of Pel rather than Psl include mutations in *bifA* [36], *rbdA* [37], *oprF* [38] and *ladS* [39]. Hence, overall our observations indicate that in non-CF BE chronic lung infections, the Pel and alginate exopolysaccharides are favoured over Psl.

Other common loss-of-function mutations (in *pilJ*, *chpA* and *fimV*) are implicated in lost or amended twitching motility, an adaptation also seen both in CF [8] and in an artificial sputum biofilm model [40], suggesting that this may be an adaptation related to the viscosity of the sputum environment.

Our study represents the first comparative genomics analysis of multiple *P. aeruginosa* isolates associated with chronic lung infections of non-CF bronchiectasis patients. Although a larger, more targeted study, analysing greater numbers of isolates per sample, would be needed to determine the true prevalence of multilineage infections, this observation does suggest that it is common for multiple *P. aeruginosa* lineages to coexist in bronchiectasis infections. Our study also demonstrates that within-sample diversity can be comparable in scale to the genetic variations that occur between isolates from different patients attending the same centre. These observations suggest that there is an urgent need for more detailed and larger scale longitudinal studies in non-CF patients, and for surveillance that captures the diversity within centres and would identify cross-infection or common source acquisition events earlier, allowing measures to be taken in order to minimise the spread of this important pathogen.

## Supplementary material

10.1183/13993003.02108-2016.Supp1 **Please note:** supplementary material is not edited by the Editorial Office, and is uploaded as it has been supplied by the author.
Supplementary material ERJ-02108-2016_Supplement
Figure S1. Core genome SNP phylogeny showing the distribution of bronchiectasis isolates. The figure shows analysis of the genomes of all bronchiectasis isolates used in this study (highlighted in blue) alongside 331 genomes from Kos *et al.* [14] and the genomes of commonly studied strains PAO1 (labelled PAO1107), PA14 (UCBPPPA14109), PA7 and LESB58. Line colours indicate the two major clusters of *P. aeruginosa* (I, green; II, blue) as well as those isolates not clustering in the two main groups (red). ERJ-02108-2016_Figure_S1
Table S4. Clone-specific deletions, relative to PAO1. ERJ-02108-2016_Table_S4
