# Supplementary material for: Pseudomonas aeruginosa adaptation and diversification in the non-cystic fibrosis bronchiectasis lung
Source: Eur Respir J. 2017 Apr 27;49(4):1602108. doi: 10.1183/13993003.02108-2016 (PMC5898933; doi:10.1183/13993003.02108-2016)
Supplement: Supplementary file 1 [file ERJ-02108-2016_Supplement.pdf]

Online Supplementary Material for:

Pseudomonas aeruginosa adaptation and diversification in the non-Cystic Fibrosis bronchiectasis lung

Yasmin Hilliam, Matthew P. Moore, Iain L. Lamont, Diana Bilton, Charles S. Haworth, Juliet Foweraker, Martin J. Walshaw, David Williams, Joanne L. Fothergill, Anthony De Soyza, Craig Winstanley

Supplementary Methods

Isolation of *P. aeruginosa* from sputum samples

All samples were cultured for routine quantitative microbiology. Sputum was homogenised with equal parts of 0.1% (v/v) dithiothreitol, and the homogenised sample was diluted in sterile distilled water to 1 in 200 and 1 in 10,000. Both dilutions were spread by Whitley Automatic Spiral Plater (WASP) onto agar plates. Cultures on Columbia blood agar, chocolate agar and cysteine lactose electrolyte deficient agar (CLED) were incubated at 37°C in 5% (v/v) CO_2 for up to 48 h. Cultures on Pseudomonas agar plus CFC supplement (PCFC) were incubated at 37°C in air for up to 48 h and then checked for small colony variants (SCV) after incubating on the bench for up to a further 48 h. Colony forming unit (CFU) representatives of up to four colonial morphotypes were sub cultured and stored in 15% (v/v) Glycerol for archiving at -80°C.

DNA preparation and genome sequencing

Genomic DNA was extracted from isolates using a Promega Wizard Genomic DNA Purification Kit, quantified using a Qubit 3.0 fluoromiter (Qubit dsDNA broad range assay kit, Life Technologies) and tested for purity using a NanoDrop 1000 spectrophotometer (Thermo Scientific). Library preparation and whole-genome shotgun sequencing was performed by the Centre for Genomic Research at the University of Liverpool, UK using Illumina short read sequencing technology. Shotgun libraries were prepared from the normalised samples using TruSeq Nano library preparation kit. Following library preparation, paired-end sequencing (2 x 100 bp) was performed by multiplexing into one lane of the Illumina HiSeq platform and sequenced with SBS V4 chemistry.

Following processing, the raw Fastq files were trimmed for the presence of Illumina adapter sequences using Cutadapt version 1.2.1 [1]. The option –O 3 was used so that the 3′ end of any reads which match the adapter sequence for 3 bp or more were trimmed. The reads were further trimmed using Sickle (https://github.com/najoshi/sickle) version 1.200 with a minimum window quality score of 20. Reads shorter than 10 bp after trimming were removed. If only one read of a pair passed this filter, it was included in the R0 file, with files R1 and R2 containing corresponding paired-end sequences. Quality filtered and adapter trimmed short reads were *de novo* assembled and scaffolded using the A5 MiSeq assembler [2]. Genome assembly quality metrics such as N50, largest contig, and overall number of contigs were produced using QUAST [3].

Core genome SNP phylogeny

The core genome was extracted using Panseq [4] and was defined as 500 bp fragments of all genomes in this study which matched with at least 85% similarity. A phylogenetic tree was approximated from core genome polymorphic sites, not including gaps or ambiguous bases by maximum likelihood with inner node bootstrap (n = 1000) and 10 discrete gamma

categories. All phylogenetic analyses were performed using MEGA6 [5] and visualised using the iTOL software [6]. Long branches were reduced for clarity.

Multilocus Sequence Typing

MLST profiles were extracted based on the pubMLST *Pseudomonas aeruginosa* scheme (http://pubmlst.org/paeruginosa/) using a specific tool (https://github.com/tseemann/mlst). It was not possible to extract complete MLST profiles from all genomes. In the context of this study, a lineage is defined on the basis of MLST profile and core genome SNP phylogeny.

Whole genome pairwise comparisons

Pairwise comparisons between assembled genomes were performed using MUMmer 3.0 [7]. Any positions in the alignment with ambiguous nucleotides were removed.

Read mapping and variant calling

All genome short read files (*fastq*) were mapped to reference genome PAO1 [8] using bwa-075a (mem) [9] producing a sequence alignment map (*sam*) files which were converted to binary alignment map files (*bam*) using picard tools-1.85 (https://broadinstitute.github.io/picard/). The reference genome was first indexed and sorted using bwa and SAMtools [10] respectively and a sequence dictionary created using picard tools-1.85. Variants were called following the Genome Analysis Toolkit (GATK) best practices workflow, as follows: duplicates were marked, the *bam* file indexed and sorted with picard tools-1.85, realignment targets created, INDELs realigned with GATK-3.3 [11] and variants called with the HaplotypeCaller module. Variants were filtered using vcffilter (https://github.com/vcflib/vcflib) with the standard parameters (DP >9, QUAL >10).

Resulting variant call files (*vcf*) were annotated to predict functional outcomes of variants compared with PAO1 genes using SnpEff [12]. Using SAMtools depth any variants within genes to which short reads had not aligned to 100% of its length were excluded from further analysis, leaving substitutions and short insertions or deletions (INDELs) and eliminating genes from functional variant analysis to which reads did not fully align due to sequencing error (lack of coverage) or genuine large deletions/complete absence.

Loss of function mutations

Predicted loss of function mutations were inferred to have been acquired independently in the population if they differed by position or type between genomes. Where the position and type were the same they were inferred to be shared; in genes where a mutation was shared with other genomes, further loss of function mutations were assumed to have been acquired since the common, ancestral acquisition of the shared mutation.

Supplementary Tables:

Supplementary Table S1. Bacterial isolates used in this study. ST refers to the designated multilocus sequence type. *novel shared MLST and they cluster according to SNP phylogeny. Isolates C21-C23 cluster together according to core genome SNP phylogeny. Isolates included in the subset of 99 genomes are highlighted in red. The Mutator column indicates the presence of mutations associated with hypermutability (INDEL, causing a frameshift or STOP, introduction of a stop codon). Of the 11 isolates carrying such a mutation, all but two (B113 and C78) were confirmed as having a hypermutable phenotype using assays reported previously [13]. Isolates labelled with the same letter from [a] to [i] were considered to be from the same lineage as each other on the basis of incomplete MLST profiles (see Table S2) and clustering by core genome SNP phylogeny (see Figure S1).

Isolate ID	Center	Date	Patient	ST	Mutator
A1	1	09/10/2014	1	17	
A2	1	16/10/2014	2	207	
A3	1	10/10/2014	3	252	
A4	1	10/10/2014	3	252	
A5	1	10/10/2014	3	252	
B3	12	01/11/2008	8	281	
B16	12	25/11/2013	9	253	
B34	12	03/09/2014	11	179	
B37	12	18/01/2012	12	-	
B62	12	03/09/2014	15	-	
B113	12	23/10/2014	18	1328	*mutL* (INDEL)
B114	12	11/04/2012	19	198	
B199	12	18/10/2011	32	1182	
A12	1	14/11/2014	35	179	
C2	4	14/10/2009	36	253	
C3	4	25/02/2010	37	260	
C4	4	25/02/2010	37	- [a]	
C5	4	25/02/2010	37	260 [a]	
C6	4	03/03/2010	38	244	
C7	4	23/03/2010	39	244	
C10	4	16/04/2010	40	244	
C8	4	16/04/2010	40	244	

C9	4	16/04/2010	40	244	
C11	4	19/08/2010	41	282	
C12	4	20/08/2010	42	282	
C13	4	20/08/2010	42	27 [b]	
C14	4	20/08/2010	42	27 [b]	
C15	4	20/08/2010	42	27 [b]	
C16	4	20/08/2010	42	- [b]	
C17	4	20/08/2010	42	- [b]	
C18	4	15/04/2011	43	-	*mutL* (STOP)
C20	4	01/07/2011	44	878	
C21	15	14/04/2009	45	- [c]	
C22	15	14/04/2009	45	- [c]	
C23	15	14/04/2009	45	- [c]	
C25	15	20/05/2009	46	253	
C29	15	03/06/2009	48	252	
C30	15	04/06/2009	49	252	
C31	15	25/08/2009	50	- [d]	*mutL* (INDEL)
C32	15	25/08/2009	50	- [d]	
C33	15	25/08/2009	50	- [d]	
C36	15	21/05/2010	52	253	
C42	15	12/07/2010	54	309	
C43	15	28/07/2010	55	108	
C44	15	28/07/2010	55	108	
C45	15	28/07/2010	55	108	
C49	15	22/02/2011	58	395	
C51	15	23/02/2011	59	683	
C54	15	15/06/2011	61	1342	
C55	5	26/06/2009	62	- [e]	
C56	5	26/06/2009	62	- [e]	
C57	5	26/06/2009	62	- [e]	
C58	5	26/06/2009	62	- [e]	
C59	5	26/06/2009	62	- [e]	
C60	5	26/06/2009	62	- [e]	
C61	5	17/11/2009	63	620	
C63	3	02/09/2009	64	27	
C64	3	25/11/2009	65	274	
C65	3	25/11/2009	65	274	
C66	3	25/11/2009	65	274	
C67	3	25/11/2009	65	274	
C68	3	25/11/2009	65	274	
C69	3	12/05/2010	66	-	

C71	9	04/09/2009	67	968	
C73	9	05/11/2010	68	17	
C74	9	03/12/2010	69	1202	*mutL* (STOP)
C76	2	12/05/2009	70	253	
C77	2	03/07/2009	71	308	
C78	2	14/07/2009	72	840	*mutL* (INDEL)
C79	2	14/07/2009	72	620[f]	*mutL* (STOP)
C80	2	14/07/2009	72	620[f]	
C81	2	14/07/2009	72	620[f]	
C82	2	14/07/2009	72	620[f]	
C83	2	14/07/2009	72	-[f]	
C84	2	14/07/2009	72	620[f]	
C85	2	06/08/2009	73	-[g]	
C86	2	06/08/2009	73	308	
C87	2	06/08/2009	73	179[g]	
C88	2	15/12/2009	74	1251	
C89	2	25/03/2010	75	1239	
C91	2	13/01/2011	76	253	
C92	2	01/02/2011	77	252	
C94	10	03/07/2009	78	395	
C95	10	29/07/2009	79	253	
C96	10	29/07/2009	79	253	
C97	10	29/07/2009	79	253	
C98	10	29/07/2009	79	253	
C99	10	29/07/2009	79	253	
C100	10	13/10/2009	80	612	
C101	10	21/10/2009	81	-[h]	
C102	10	21/10/2009	81	-[h]	
C103	10	21/10/2009	81	-[h]	
C104	13	16/05/2009	82	179	
C105	13	25/07/2009	83	840	
C106	13	11/08/2009	84	-[i]	
C107	13	11/08/2009	84	253	
C108	13	11/08/2009	84	179[i]	
C109	13	11/08/2009	85	840	
C110	13	11/08/2009	85	179	
C111	13	11/08/2009	85	179	
C112	13	11/08/2009	85	179	
C114	13	05/12/2009	86	179	
C115	13	05/12/2009	86	179	
C116	13	04/06/2010	87	871	

C117	13	04/06/2010	87	871	
C118	13	04/06/2010	87	871	
C119	7	23/01/2010	88	-	
C120	7	29/01/2010	89	-	
C123	7	02/04/2010	90	27	*mutS* (INDEL)
C124	7	08/04/2010	91	1753	
C125	7	29/04/2010	92	253	
C126	7	29/04/2010	92	253	
C127	7	29/04/2010	92	164	*mutS* (INDEL)
C128	7	29/04/2010	92	164	
C129	7	29/04/2010	92	871	*mutL* (INDEL)
C131	7	08/05/2010	93	253	
C133	7	28/05/2010	94	253	*mutS* (INDEL)
C134	7	19/12/2010	95	253	*mutS* (INDEL)
C135	14	02/11/2009	96	160	
C137	14	16/04/2010	97	260	
C139	14	08/09/2010	98	2102	
C141	14	01/10/2010	99	2102	
C142	14	11/02/2011	100	252	
C143	8	08/07/2010	101	253	
C144	8	08/07/2010	101	253	
C145	8	08/07/2010	101	253	
C146	8	25/06/2010	102	395	
C147	8	25/06/2010	102	395	
C148	8	25/06/2010	102	395	
C149	8	22/03/2011	103	108	
C150	6	27/08/2010	104	253	
C151	6	03/03/2011	105	1244	
C153	6	07/04/2011	106	155	
C155	11	04/12/2010	107	1211	
C156	16	16/11/2010	108	260	
C158	16	03/12/2010	109	155	
C159	16	09/12/2010	110	260	
C160	16	09/12/2010	111	1244	
C161	16	03/03/2011	112	110	
C164	16	08/12/2010	113	-	
C167	16	21/04/2011	114	296	
C168	16	21/12/2010	115	17	
A19	1	16/12/2014	120	-	
A163	1	19/05/2015	137	146	
A36	1	17/02/2015	137	146	

A46	1	07/04/2015	147	17	
A48	1	07/04/2015	147	17	
A52	1	07/04/2015	147	17	
A53	1	07/04/2015	147	17	
A54	1	07/04/2015	147	17	
A55	1	07/04/2015	147	17	
A56	1	07/04/2015	147	17	
A58	1	07/04/2015	147	17	
A60	1	07/04/2015	147	17	
A70	1	07/04/2015	147	17	
A71	1	07/04/2015	147	17	
A72	1	07/04/2015	147	17	
A73	1	07/04/2015	147	17	
A75	1	07/04/2015	147	17	
A76	1	07/04/2015	147	17	
A100	1	07/04/2015	148	17	
A106	1	07/04/2015	148	175	
A107	1	07/04/2015	148	175	
A77	1	07/04/2015	148	175	
A78	1	07/04/2015	148	17	
A80	1	07/04/2015	148	175	
A81	1	07/04/2015	148	17	
A82	1	07/04/2015	148	17	
A85	1	07/04/2015	148	175	
A86	1	07/04/2015	148	175	
A90	1	07/04/2015	148	175	
A91	1	07/04/2015	148	175	
A92	1	07/04/2015	148	175	
A95	1	07/04/2015	148	175	
A97	1	07/04/2015	148	175	
A119	1	15/05/2015	149	667	
A122	1	15/05/2015	149	667	
A123	1	15/05/2015	149	667	
A126	1	15/05/2015	149	667	
A130	1	15/05/2015	149	667	
A134	1	15/05/2015	149	667	
A137	1	15/05/2015	149	667	
A141	1	15/05/2015	149	667	
A144	1	15/05/2015	149	667	
A147	1	15/05/2015	149	667	
A148	1	15/05/2015	149	667	

A151	1	15/05/2015 149	667	
A154	1	15/05/2015 149	667	
A156	1	15/05/2015 149	667	

Supplementary Table S2. MLST profiles of isolates where incomplete profiles were obtained or the MLST profile was novel.

Isolate	ST	Closest ST	*Pseudomonas aeruginosa* MLST loci allele numbers						
			acs	*aro*	*gua*	*mut*	*nuo*	*pps*	*trp*
**A19**	NF	92 or 261	105	5	30	-	1	4	14
**B37**	Novel		107	4	3	27	12	7	128
**B62**	NF	1404	16	-	6	3	4	7	1
**C101**	NF	303 (4 loci)	16	-	12	18	3	4	9
**C102**	NF	304 (4 loci)	16	-	12	18	3	4	9
**C103**	NF	305 (4 loci)	16	-	12	18	3	4	9
**C106**	NF	156,179,353,1494 (6 loci)	-	27	28	3	4	13	7
**C119**	Novel		5	1	109	3	1	1	47
**C120**	Novel		17	5	11	5	4	29	2
**C164**	NF	1240,1985 (4 loci)	28	5	46	5	1	-	61
**C16**	NF	27,120,2314 (5 loci)	6	-	6	113	4	6	7
**C17**	NF	27,120,2314 (5 loci)	6	-	6	113	4	6	7
**C18**	NF	158,179,180,1496,2063,2109 (6 loci)	36	27	28	-	4	13	7
**C21**	Novel		22	6	1	3	1	76	1
**C22**	Novel		22	6	1	3	1	76	1
**C23**	Novel		22	6	1	3	1	76	1
**C31**	NF	155,677,1276 (5 loci)	28	5	36	-	3	13	7

**C32**	NF	155,677,1276 (5 loci)	28	5	36	-	3	13	7
**C33**	NF	155,677,1276 (5 loci)	28	5	36	-	3	13	7
**C4**	NF	260 (6 loci)	14	5	-	7	4	13	7
**C55**	Novel		28	5	11	11	15	75	1
**C56**	Novel		28	5	11	11	15	75	1
**C57**	Novel		28	5	11	11	15	75	1
**C58**	Novel		28	5	11	11	15	75	1
**C59**	Novel		28	5	11	11	15	75	1
**C60**	Novel		28	5	11	11	15	75	1
**C69**	NF	1707, 2055 (6 loci)	16	24	1	149	4	-	19
**C83**	NF	620 (6 loci)	9	7	63	13	8	-	8
**C85**	NF	156, 179, 353, 1494 (6 loci)	-	27	28	3	4	13	7

Table S3. Clonal lineages isolated from multiple patients within individual centres. Sets of isolates from different patients attending the same centre are grouped by clonal lineage. For each such group, whole genome pairwise comparisons were carried out to determine the number of variant SNPs and INDELs. *These isolates share a novel MLST profile and they cluster according to SNP phylogeny.

Isolate	Centre	Isolation date	Patient	MLST	Comparison	SNPs	INDELs
C6	4	03/03/2010	38	244	C6-C7	179	8
					C6-C8	3790	79
C7	4	23/03/2010	39	244	C6-C9	3733	74
					C6-C10	3833	62
C8	4	16/04/2010	40	244	C7-C8	3736	82
					C7-C9	3714	75
C9	4	16/04/2010	40	244	C8-C9	281	5
					C7-C10	3929	65
C10	4	16/04/2010	40	244	C8-C10	603	14
					C9-C10	515	11
C11	4	19/08/2010	41	282	C11-C12	340	8
C12	4	20/08/2010	42	282			
C29	15	03/06/2009	48	252	C29-C30	168	3
C30	15	04/06/2009	49	252			
C25	15	20/05/2009	46	253	C25-C36	3428	43
C36	15	21/05/2010	52	253			
C91	2	13/01/2011	76	253	C76-C91	846	5
C76	2	12/05/2009	70	253			
C77	2	03/07/2009	71	308	C77-C86	277	1
C86	2	06/08/2009	73	308			
C105	13	25/07/2009	83	840	C105-C109	131	8
C109	13	11/08/2009	85	840			
C104	13	16/05/2009	82	179	C104-C108	10,551	114
					C104-C110	2863	39
					C104-C111	2765	52
C108	13	11/08/2009	84	179	C104-C112	1913	25
					C104-C114	6795	96
					C104-C115	6972	105
C110	13	11/08/2009	85	179	C108-C110	4780	78
					C108-C111	4852	66
					C108-C112	3780	62

C111	13	11/08/2009	85	179	C108-C114	10,963	136
					C108-C115	10,833	133
					C110-C111	176	8
C112	13	11/08/2009	85	179	C110-C112	198	7
					C110-C114	3115	40
					C110-C115	3107	45
C114	13	05/12/2009	86	179	C111-C112	148	4
					C111-C114	2838	43
					C111-C115	2789	57
C115	13	05/12/2009	86	179	C112-C114	1941	29
					C112-C115	1895	31
					C114-C115	281	4
C125	7	29/04/2010	92	253	C125-C126	736	27
					C125-C131	1159	22
C126	7	29/04/2010	92	253	C125-C133	872	22
					C125-C134	817	21
C131	7	08/05/2010	93	253	C126-C131	9636	99
					C126-C133	5847	71
C133	7	28/05/2010	94	253	C126-C134	5883	70
					C131-C133	3486	40
C134	7	19/12/2010	95	253	C131-C134	3400	38
					C133-C134	330	4
C156	16	16/11/2010	108	260	C156-C159	160	3
C159	16	09/12/2010	110	260			
C139	14	08/09/2010	98	ST2102	C139-C141	177	3
C141	14	01/10/2010	99	ST2102			

Supplementary Table S4 and Figure S1 are provided in additional files:

Table S4. Clone-specific deletions, relative to PAO1

Figure S1. Core genome SNP phylogeny showing the distribution of bronchiectasis isolates. The figure shows analysis of the genomes of all bronchiectasis isolates used in this study (highlighted in blue) alongside 331 genomes from Kos et al. [14] and the genomes of commonly studied strains PAO1 (labelled PAO1107), PA14 (UCBPPPA14109), PA7 and LESB58. Line colours indicate the two major clusters of *P. aeruginosa* (I, green; II, blue) as well as those isolates not clustering in the two main groups (red).

Figure S2. Distribution of loss of function mutations. For the genes listed in Table 2, where the number of independent occurrences of a loss of function mutation was equal to or greater than five, the Figure indicates the number of isolates carrying mutations in 0, 1, 2, 3, 4, 5 or 6 of these genes.

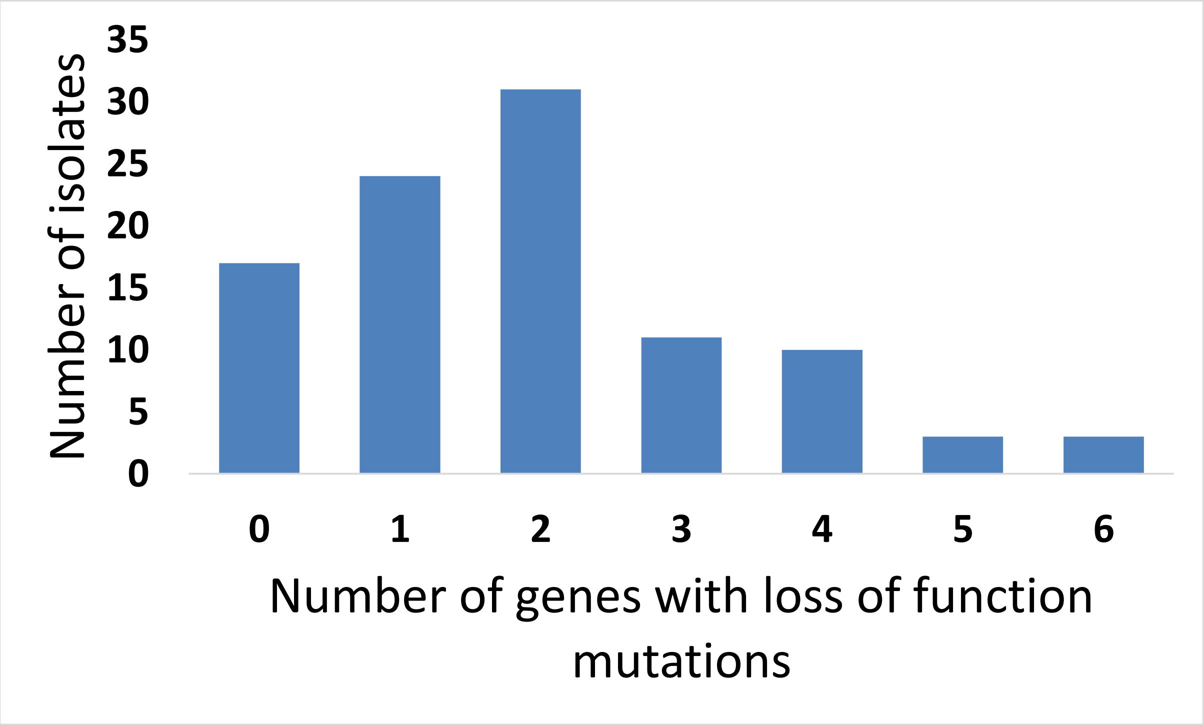

Table S5. Full list of loss of function mutations identified by variant calling. Available via the Figshare link https://figshare.com/s/ff426bae75ee64804aa1

Table S6. Loss of function mutations present in each bronchiectasis isolate genome. For the genes listed in Table 2, where the number of independent occurrences of a loss of function mutation was equal to or greater than five, the genes carrying such mutations are shown for each of the bronchiectasis isolates. 17 of the 99 isolates carried none of these mutations. It is worth noting that isolates found co-infecting individual patients did not share the same mutation profile (C125, C127

and C129 in patient 92; C12 and C13 in patient 42; C78 and C79 in patient 72; C86 and C87 in patient 73; C107 and C108 in patient 84; C109 and C110 in patient 85; A100 and A106 in patient 148).

Isolate	Number of Table2 genes with loss of function mutations	Mutations					
C125	6	PA4469	mexS	ladS	rbdA	betT2	mexA
C74	6	PA4469	mexS	chpA	mutL	pchE	mexB
C87	6	PA4469	bifA	pchE	ladS	chpA	mexA
C123	5	rbdA	PA4469	pchE	mexS	oprM	
C116	5	PA4469	mucA	mexB	oprF	pchE	
C88	5	mucA	pcoA	betT2	oprF	mexA	
C100	4	rbdA	PA4469	mucA	chpA		
C79	4	rbdA	PA4469	mucA	mutL		
C133	4	rbdA	mucA	pchE	oprM		
C95	4	rbdA	mucA	oprM	pcoA		
B16	4	rbdA	mucA	mexB	fimV		
C21	4	PA4469	mucA	mexB	pcoA		
C31	4	PA4469	mucA	mutL	fimV		
C110	4	PA4469	mucA	oprF	mexA		
C108	4	mexB	chpA	mucA	betT2		
C129	4	mucA	mexB	oprF	gmd		
C134	3	rbdA	mucA	oprM			
C143	3	rbdA	mucA	mexB			
C2	3	rbdA	mexB	chpA			
A119	3	PA4469	oprF	pcoA			
A46	3	PA4469	mucA	oprF			
C63	3	betT2	ladS	fimV			
C114	3	mexB	pchE	betT2			
A163	3	mucA	mexB	gmd			
C149	3	mucA	pchE	oprM			
C55	3	mucA	pilJ	oprF			
B113	3	pilJ	mutL	pcoA			
C124	2	PA0054	mucA				
C146	2	PA0054	mexA				
C49	2	PA0054	betT2				
C51	2	PA0054	rbdA				
B114	2	rbdA	mexB				
C25	2	rbdA	mexB				
C36	2	rbdA	mucA				
C86	2	rbdA	mexA				

C20	2	rbdA	pilJ
C137	2	rbdA	PA4469
C6	2	PA4469	mexS
C7	2	PA4469	mexS
C135	2	mucA	mexS
C61	2	PA4469	mexA
C42	2	PA4469	oprF
A100	2	PA4469	fimV
B62	2	PA4469	pilJ
C131	2	betT2	ladS
B34	2	bifA	betT2
C12	2	bifA	betT2
C11	2	bifA	betT2
A1	2	bifA	ladS
B3	2	fimV	pcoA
C167	2	mexA	gmd
C3	2	pilJ	mexB
A2	2	mucA	mexB
C153	2	mexB	oprF
C158	2	mexB	oprF
C161	2	mucA	pcoA
C18	2	oprM	mutL
C155	2	pilJ	oprM
C94	1	PA0054	
C120	1	rbdA	
A3	1	rbdA	
C77	1	rbdA	
C91	1	rbdA	
C69	1	rbdA	
C156	1	rbdA	
C159	1	rbdA	
C107	1	betT2	
C119	1	bifA	
A106	1	bifA	
C43	1	bifA	
B199	1	gmd	
C89	1	gmd	
C168	1	gmd	
A12	1	mexB	
C54	1	mexB	
C92	1	mucA	
C10	1	mucA	
C73	1	mucA	
C142	1	mucA	
C76	1	mucA	
B37	1	mucA	

C101	1	oprM

Table S7. The presence or absence of virulence genes amongst the non-CF BE isolate genomes. Available via the Figshare link https://figshare.com/s/626a59cfd94b13e5cf71

References

1. Martin M. Cutadapt removes adapter sequences from high-throughout sequencin reads. *EMBnet J* 2011: 17(1): 10-12.
2. Coil D, Jospin G, Darling AE. A5-miseq: an updated pipeline to assemble microbial genomes from Illumina MiSeq data. *Bioinformatics* 2015: 31(4): 587-589.
3. Gurevich A, Saveliev V, Vyahhi N, Tesler G. QUAST: quality assessment tool for genome assemblies. *Bioinformatics* 2013: 29(8): 1072-1075.
4. Laing C, Buchanan C, Taboada EN, Zhang Y, Kropinski A, Villegas A, Thomas JE, Gannon VP. Pan-genome sequence analysis using Panseq: an online tool for the rapid analysis of core and accessory genomic regions. *BMC bioinformatics* 2010: 11: 461.
5. Tamura K, Stecher G, Peterson D, Filipski A, Kumar S. MEGA6: Molecular Evolutionary Genetics Analysis version 6.0. *Mol Biol Evol* 2013: 30(12): 2725-2729.
6. Letunic I, Bork P. Interactive Tree Of Life (iTOL): an online tool for phylogenetic tree display and annotation. *Bioinformatics* 2007: 23(1): 127-128.
7. Kurtz S, Phillippy A, Delcher AL, Smoot M, Shumway M, Antonescu C, Salzberg SL. Versatile and open software for comparing large genomes. *Genome biology* 2004: 5(2): R12.
8. Stover CK, Pham XQ, Erwin AL, Mizoguchi SD, Warrener P, Hickey MJ, Brinkman FS, Hufnagle WO, Kowalik DJ, Lagrou M, Garber RL, Goltry L, Tolentino E, Westbrock-Wadman S, Yuan Y, Brody LL, Coulter SN, Folger KR, Kas A, Larbig K, Lim R, Smith K, Spencer D, Wong GK, Wu Z, Paulsen IT, Reizer J, Saier MH, Hancock RE, Lory S, Olson MV. Complete genome sequence of Pseudomonas aeruginosa PA01, an opportunistic pathogen. *Nature* 2000: 406(6799): 959-964.
9. Li H, Durbin R. Fast and accurate short read alignment with Burrows-Wheeler transform. *Bioinformatics* 2009: 25(14): 1754-1760.
10. Li H, Handsaker B, Wysoker A, Fennell T, Ruan J, Homer N, Marth G, Abecasis G, Durbin R, Genome Project Data Processing S. The Sequence Alignment/Map format and SAMtools. *Bioinformatics* 2009: 25(16): 2078-2079.
11. McKenna A, Hanna M, Banks E, Sivachenko A, Cibulskis K, Kernytsky A, Garimella K, Altshuler D, Gabriel S, Daly M, DePristo MA. The Genome Analysis Toolkit: a MapReduce framework for analyzing next-generation DNA sequencing data. *Genome research* 2010: 20(9): 1297-1303.
12. Cingolani P, Platts A, Wang le L, Coon M, Nguyen T, Wang L, Land SJ, Lu X, Ruden DM. A program for annotating and predicting the effects of single nucleotide polymorphisms, SnpEff: SNPs in the genome of Drosophila melanogaster strain w1118; iso-2; iso-3. *Fly (Austin)* 2012: 6(2): 80-92.
13. Mowat E, Paterson S, Fothergill JL, Wright EA, Ledson MJ, Walshaw MJ, Brockhurst MA, Winstanley C. Pseudomonas aeruginosa population diversity and turnover in cystic fibrosis chronic infections. *AmJRespirCrit Care Med* 2011: 183(12): 1674-1679.
14. Kos VN, Deraspe M, McLaughlin RE, Whiteaker JD, Roy PH, Alm RA, Corbeil J, Gardner H. The resistome of Pseudomonas aeruginosa in relationship to phenotypic susceptibility. *Antimicrobial agents and chemotherapy* 2015: 59(1): 427-436.
