# Supplementary figures and images for: Pseudomonas aeruginosa adaptation and diversification in the non-cystic fibrosis bronchiectasis lung
Source: Eur Respir J. 2017 Apr 27;49(4):1602108. doi: 10.1183/13993003.02108-2016 (PMC5898933; doi:10.1183/13993003.02108-2016)

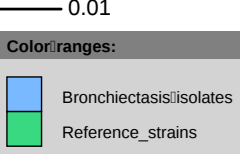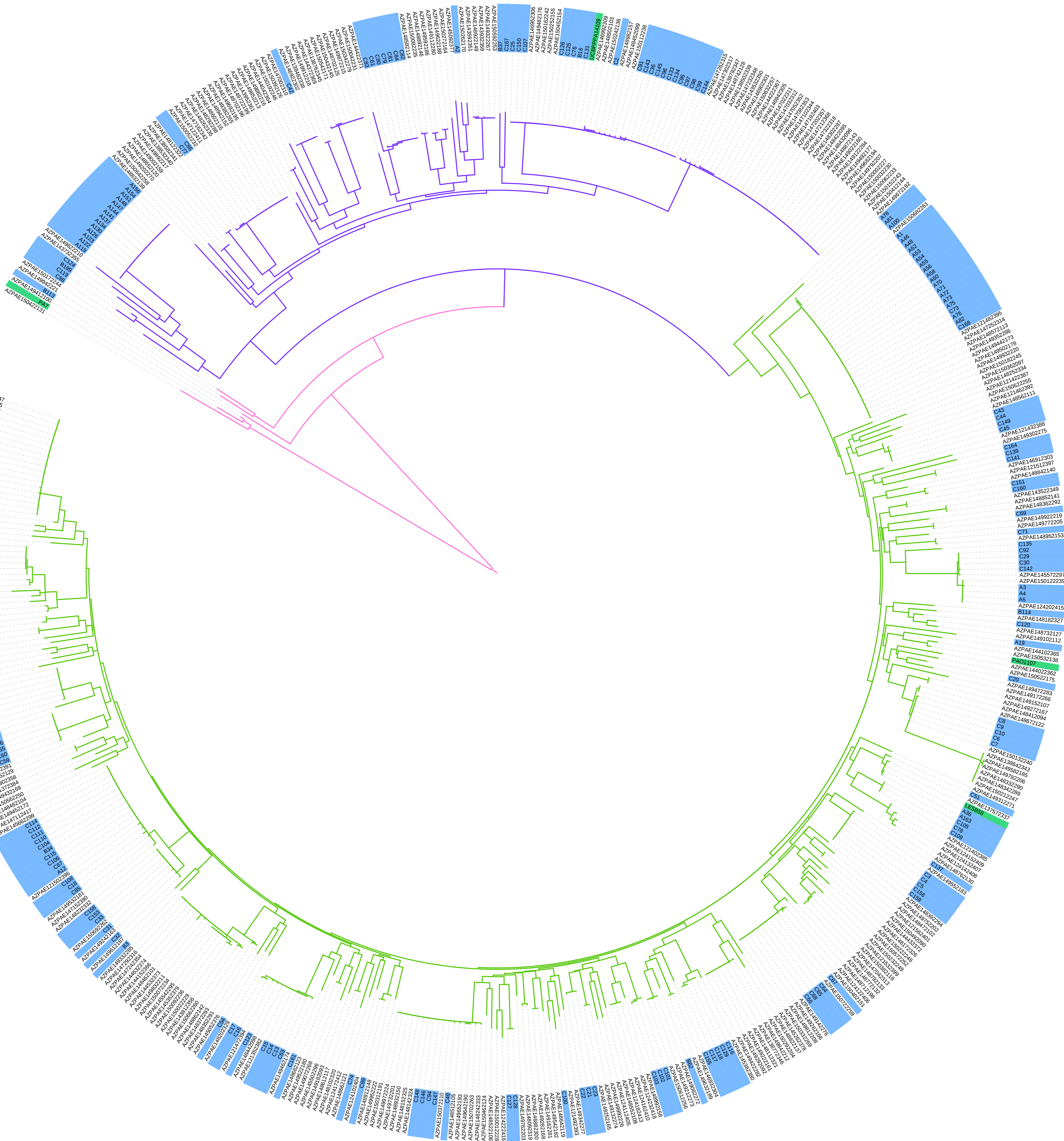

Supplement: Supplementary file 2 [file ERJ-02108-2016_Figure_S1.pdf]
