# Supplementary material for: Pseudomonas aeruginosa adaptation and diversification in the non-cystic fibrosis bronchiectasis lung
Source: Eur Respir J. 2017 Apr 27;49(4):1602108. doi: 10.1183/13993003.02108-2016 (PMC5898933; doi:10.1183/13993003.02108-2016)
Supplement: Supplementary file 3 [file ERJ-02108-2016_Table_S4.pdf]

Table S4. Clone-specific deletions relative to PAO1.

Isolate*	Deleted DNA† From	To	Size of deletion (bp)	Deleted Genes‡ From	To
A12	2448472	2723305	274833	PA2226	PA2427
A119	2443094	2624360	181189	PA2221	PA2373
A119	5455322	5497400	42078	PA4857	PA4900
A163	2543016	2705463	162447	PA2305	PA2421
A19	2420900	2721500	300600	PA2201	PA2425
A78	2439040	2574546	135506	PA2218	PA2333
B34	1979190	2015165	35975	PA1820	PA1856
B62	2461847	2686720	224873	PA2237	PA2402
C100	2443095	2724005	280910	PA2221	PA2428
C101	2452047	2578374	126327	PA2229	PA2335
C119	2184340	2247131	62791	PA1997	PA2053
C119	2487183	2689945	202762	PA2258	PA2406
C125	2439064	2729482	290418	PA2218	PA2432
C135	2443095	2588618	145523	PA2221	PA2343
C137	2453340	2560360	107020	PA2231	PA2321
C155	2440000	2585490	145490	PA2218	PA2431
C156	2439060	2475845	36785	PA2218	PA2249
C164	2248926	2451500	202574	PA2055	PA2228
C164	2793790	2810695	16905	PA2475	PA2494
C164	3914150	3930740	16590	PA3497	PA3514
C21	2439057	2713422	274365	PA2218	PA2424
C22	2209250	2442066	232816	PA2018	PA2220
C4	111095	211425	100330	PA0091	PA0781
C4	2439055	2666000	226945	PA2218	PA2400
C44	2443095	2615075	171980	PA2221	PA2365
C5	3841807	3873962	32155	PA3434	PA3463
C51	2071372	2199654	128282	PA1900	PA2010
C54	762796	789380	26584	PA0691	PA0717
C54	2410960	2478550	67590	PA2191	PA2251
C54	2628400	2817536	189136	PA2377	PA2500
C61	4005650	4016519	10869	PA3573	PA3584
C6	2448920	2689471	240551	PA2227	PA2405
C73	2460955	2584136	123181	PA2236	PA2399
C87	2461666	2606428	144762	PA2237	PA2359
C87	2787221	2828217	40996	PA2469	PA2511
C96	2705835	2863924	158089	PA2422	PA2535

*For identical strains with the same deletion, only one strain is listed
†Coordinates of P. aeruginosa PAO1
‡P. aeruginosa strain PAO1 locus ID
